# Synthetic Human β Defensin-3-C15 Peptide in Endodontics: Potential Therapeutic Agent in *Streptococcus gordonii* Lipoprotein-Stimulated Human Dental Pulp-Derived Cells

**DOI:** 10.3390/ijms21010071

**Published:** 2019-12-20

**Authors:** Yeon-Jee Yoo, Hiran Perinpanayagam, Jue-Yeon Lee, Soram Oh, Yu Gu, A-Reum Kim, Seok-Woo Chang, Seung-Ho Baek, Kee-Yeon Kum

**Affiliations:** 1 Department of Conservative Dentistry, School of Dentistry, Dental Research Institute, Seoul National University, Daehak-ro 101, Chongno-Ku, Seoul 03080, Korea; duswl32@snu.ac.kr (Y.-J.Y.); shbaek@snu.ac.kr (S.-H.B.); 2 Department of Dentistry, Schulich School of Medicine & Dentistry, University of Western Ontario, London, ON N6A 5C1, Canada; hperinpa@uwo.ca; 3 Central Research Institute, Nano intelligent Biomedical Engineering Corporation (NIBEC), Seoul 03130, Korea; yeon0417@nibec.co.kr; 4 Department of Conservative Dentistry, School of Dentistry, Kyung Hee University, Seoul 02447, Korea; soram0123@naver.com (S.O.); swc2007smc@gmail.com (S.-W.C.); 5 Department of Conservative Dentistry, School of Stomatology, Shandong University, Jinan 250012, China; guyu618@126.com; 6 Department of Oral Microbiology and Immunology, School of Dentistry, Dental Research Institute and BK21 Plus Program, Seoul National University, Seoul 08826, Korea; kimareum@snu.ac.kr; 7 National Dental Care Center for Persons with Special Cares, Seoul National University Dental Hospital for Persons with Special Needs, Seoul 03080, Korea

**Keywords:** calcium hydroxide, chemokine, human beta defensin-3-C15, human dental pulp cell, *Streptococcus gordonii* lipoprotein

## Abstract

Human β defensin-3-C15, an epithelium-derived cationic peptide that has antibacterial/antifungal and immuno-regulatory properties, is getting attention as potential therapeutic agent in endodontics. This study aimed to investigate if synthetic human β defensin-3-C15 (HBD3-C15) peptides could inhibit inflammatory responses in human dental pulp cells (hDPCs), which had been induced by gram-positive endodontic pathogen. hDPC explant cultures were stimulated with *Streptococcus gordonii* lipoprotein extracts for 24 h to induce expression of pro-inflammatory mediators. The cells were then treated with either HBD3-C15 (50 μg/mL) or calcium hydroxide (CH, 100 μg/mL) as control for seven days, to assess their anti-inflammatory effects. Quantitative RT-PCR analyses and multiplex assays showed that *S. gordonii* lipoprotein induced the inflammatory reaction in hDPCs. There was a significant reduction of IL-8 and MCP-1 within 24 h of treatment with either CH or HBD3-C15 (*p* < 0.05), which was sustained over 1 week of treatment. Alleviation of inflammation in both medications was related to COX-2 expression and PGE2 secretion (*p* < 0.05), rather than TLR2 changes (*p* > 0.05). These findings demonstrate comparable effects of CH and HDB3-C15 as therapeutic agents for inflamed hDPCs.

## 1. Introduction

*Streptococcus gordonii* are Gram-positive facultative anaerobes that are frequently isolated from cases of recurrent apical periodontitis, due to their propensity to form bacterial biofilms on root canal surfaces [1]. Additionally, they can exchange genes that encode cytotoxins, adhesins, and antibiotic resistance, with *Enterococcus faecalis* [2]. These virulence features enable *S. gordonii* to play an important role in the pathogenesis of apical periodontitis.

Virulence factors in gram-positive bacteria include cell wall-associated lipoteichoic acid (LTA) and lipoproteins, which are recognized by Toll-like receptor 2 (TLR2) [3,4]. These stimulate a variety of host cells to induce pro-inflammatory cytokines and chemokines. For instance, LTA from *Staphylococcus aureus* or *Streptococcus pyogens* were shown to induce IL-8 in human peripheral blood monocytes. *S. aureus* lipoproteins were shown to induce IL-8 in human intestinal epithelial cells. Likewise, LTA, lipoproteins, and peptidoglycan from *S. gordonii* induced the pro-inflammatory cytokines IL-6 and TNFα in dendritic cells, via TLR2 [5]. Notably, the *S. gordonii* lipoprotein is reported to be a key virulence factor in inducing inflammatory responses [6,7].

**Figure 1:**
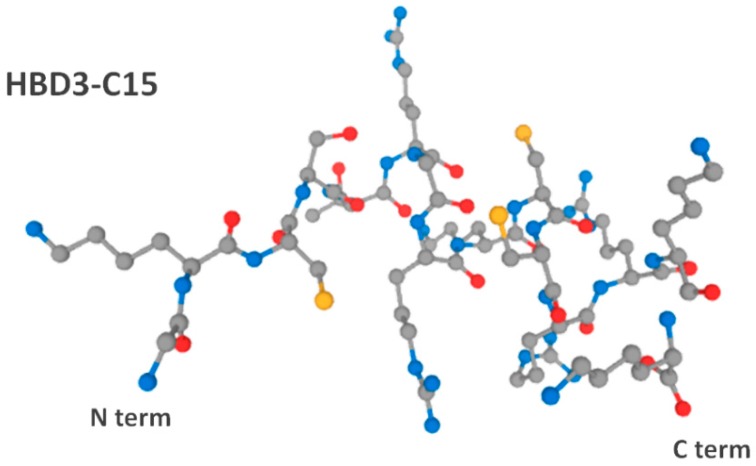
Molecular structure of synthetic human β defensin-3-C15 (HBD3-C15) peptide, prepared by F-moc-based chemical solid phase synthesis from 15 amino acids (GKCSTRGRKCCRRKK). Grey, carbon; blue, nitrogen; red, oxygen; yellow, sulfur.

To inactivate virulence factors and eliminate bacteria, intracanal medicaments are often applied to root canal systems for treating apical periodontitis. The most widely used medicament is calcium hydroxide (CH), which has antimicrobial effects that are largely due to its high pH [8]. It has been shown to suppress both lipopolysaccharide (LPS) [9] and LTA [10,11], which are virulence factors critical to Gram-negative and -positive bacteria respectively. More recently, antimicrobial peptides have been developed as potential therapeutic agents against microbial biofilms [12]. These include human β-defensin-3 (HBD3), which is a cysteine-rich cationic peptide that has strong antibacterial, antifungal and immuno-regulatory properties [13,14,15,16]. The disulfide topology that maintains tertiary structure in HBD3 is dispensable for its antimicrobial functions [17], and the C-terminal end of HBD3 contains 15 amino acids that effectively elicit antimicrobial activity [18]. A synthetic HBD3 peptide that consists of only 15 amino acids from the C-terminus (HBD3-C15, GKCSTRGRKCCRRKK), is being developed as an alternative antibiofilm agent (Figure 1) [12]. This 15-mer peptide derived from the C-terminus of HBD3 (HBD3-C15) has been shown to have considerable antimicrobial activity that is comparable to that of the full-length protein [18,19,20,21]. However, little is known about its suppressive effects on gram-positive bacterial virulence factors.

Where the inactivation or elimination of virulence factors are inadequate, the induction of pro-inflammatory mediators ensure a persistence of pulpitis and apical periodontitis [22,23]. Chemokines such as interleukin-8 (IL-8) and monocyte chemoattractant protein-1 (MCP-1) display potent chemotactic activities for human neutrophils and T lymphocytes, which in turn trigger a series of inflammatory events [24] that release enzymes causing tissue destruction [25]. Accompanying inflammatory mediators such as cyclooxygenase 2 (COX-2) stimulate vasodilation and microvascular permeability by cytoskeletal rearrangement or contraction of vascular smooth muscle [26]. Therefore, the purpose of this study was to examine the anti-inflammatory properties of HBD3-C15 in human dental pulp cells (hDPCs) that had been stimulated by *S. gordonii* derived lipoprotein, by comparing the effects of HBD3-C15 and CH.

## 2. Results

### 2.1. S. gordoni Lipoproteins Induced Inflammatory Mediators

**Figure 2:**
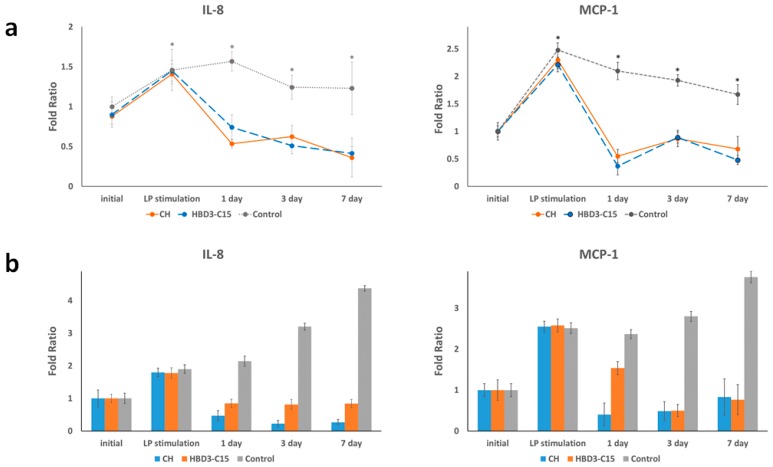
Cultured human dental pulp cells (hDPCs) experienced an inflammatory reaction after Gram-positive *S. gordonii* lipoprotein (LP)-stimulation, and then inflamed hDPCs were treated with either calcium hydroxide (CH) or HBD3-C15. Cell lysates were collected at the indicated time points, and the expressions of IL-8 and MCP-1 were analyzed by (**a**) qRT-PCR and (**b**) multiplex assay. The relative expression levels of each control group are presented.

*S. gordonii* lipoprotein induced the expression of chemokines IL-8 and MCP-1 by hDPC explant cultures (Figure 2). When hDPCs were treated with Triton X-114 extracts from *S. gordonii* that contained the bacterial lipoproteins [27], there was an immediate increase in the relative mRNA expressions of IL-8 and MCP-1, as measured by real-time RT-qPCR. The relative levels of IL-8 and MCP-1 were significantly higher (*p* < 0.05) in the *S. gordonii* lipoprotein-treated hDPCs, than in the untreated cultures.

### 2.2. HBD3-C15 and CH Suppress Inflammatory Mediators

The stimulated cells were then treated with either HBD3-C15 or CH. The medicaments were not simultaneously treated with *S. gordonii* lipoprotein, to prevent the interactions and denaturation of *S. gordonii* lipoprotein with positively charged HBD3-C15 or highly alkaline CH, and also to reenact clinical relevance in endodontics. Both HBD3-C15 and CH treatments for 1, 3, and 7 days, suppressed the expression of IL-8 and MCP-1 mRNA measured by qRT-PCR (Figure 2a), and their protein level quantified by multiplex assay (Figure 2b). There were significant and sustained reductions in IL-8 and MCP-1 mRNA gene expression and their quantified protein level, to the levels those were at or below their baselines values (*p* < 0.05). There were no significant differences between these two medicaments (*p* > 0.05). Non-medicated cells showed sustained inflammatory status, with increased protein level over time.

### 2.3. Anti-Inflammatory Effects of HBD3-C15 and CH Were not Mediated by TLR2

**Figure 3:**
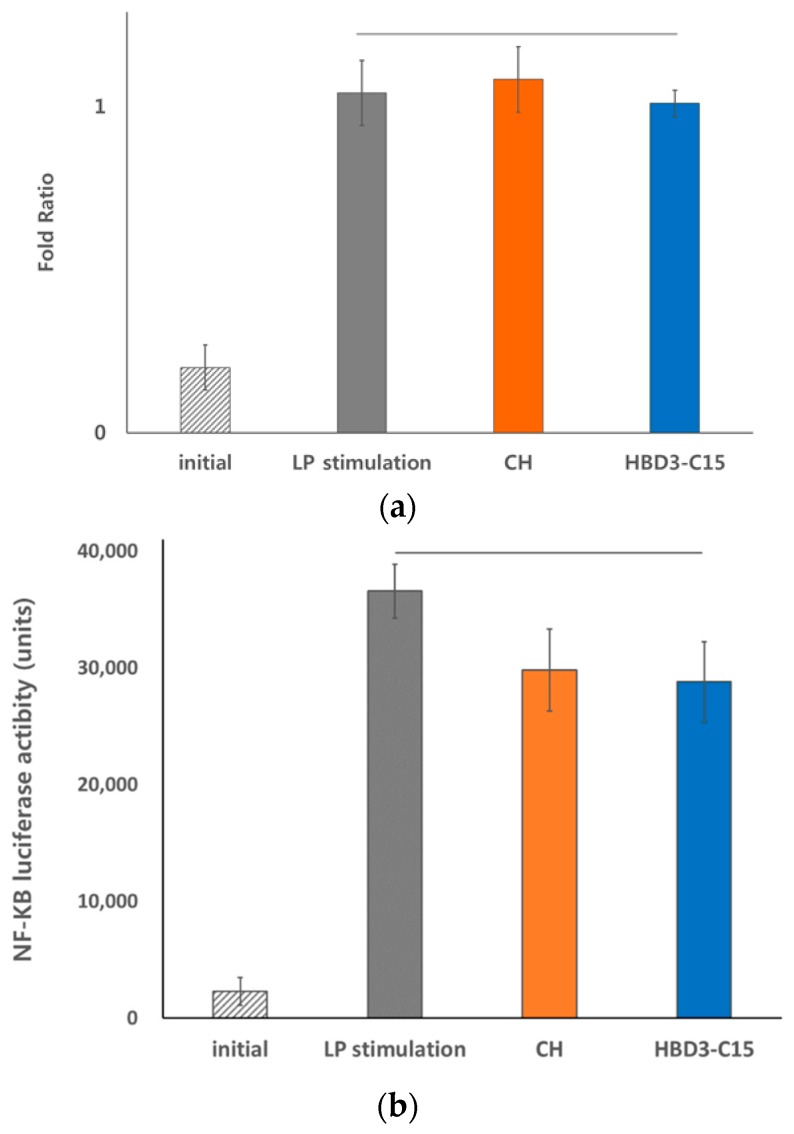
(**a**) Exogenous stimulation of *S. gordonii* lipoproteins augment TLR2 mRNA expressions in hDPCs, and were maintained after calcium hydroxide (CH) or HBD3-C15 treatment. (**b**) *S. gordonii* lipoprotein-stimulated NF-κB are mediated by TLR2. HEK-TLR2 cells (2.5 × 10^5^ cells/mL) were transfected with an NF-κB luciferase reporter plasmid using Attractene transfection reagent. After 16 h, the cells were stimulated with lipoprotein purified from *S. gordonii*, and then treated with either of CH or HBD3-C15. After the cells were lysed, a luciferease assay was conducted.

To investigate the underlying anti-inflammatory mechanism of HBD3-C15 and CH, TLR2 mRNA expression level was assessed in hDPCs. *S. gordonii* lipoproteins induced upregulated TLR2 expression in hDPCs (*p* < 0.05). However, TLR2 gene expression level were not decreased after medication (Figure 3a). To validate the result, we examined HEK292-TLR2 cell transfection and found that *S. gordonii* lipoproteins potently stimulated NF-κB in HEK292-TLR2 cells as in hDPCs, but there were no significant differences after medication (Figure 3b, *p* > 0.05).

### 2.4. Anti-Inflammatory Effects Were Related to COX-2 Expression and PGE2 Level

**Figure 4:**
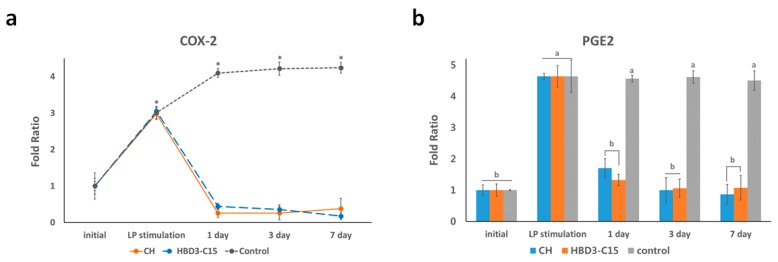
(**a**) Expression of COX-2 gene in *S. gordonii* lipoprotein-stimulated hDPCs after treatment with calcium hydroxide (CH) or HBD3-C15 at determined time points and (**b**) PGE2 secretion levels accordingly. The relative expression levels to each control group are presented.

To extend the findings to speculate on the mechanism of inflammatory status changes after treatment, we investigated the inflammatory marker COX-2 changes of inflamed hDPCs, which is known to be upregulated in pulpal inflammations [28]. It was found that the COX-2 gene expression and PGE2 level were significantly upregulated after *S. gordonii* stimulation of hDPCs (*p* < 0.05), and were significantly alleviated after 24 h of CH and HBD3-C15 treatments (*p* < 0.05), and maintained at low level (Figure 4). There was no significant differences between the two medicaments (*p* > 0.05).

## 3. Discussion

Pulp and periapical inflammation are initiated and propagated by chemokines. The pro-inflammatory chemokines IL-8 and MCP-1 are associated with the recruitment of cells to sites of inflammation. A predominant cellular source of IL-8 and MCP-1 are mononuclear phagocytes. However, IL-8 and MCP-1 can also be produced by nonimmune cells, such as fibroblasts, keratinocytes, and endothelial cells in response to either endogenous or exogenous stimuli. The cytokines produced by mononuclear cells are important mediators for chemokine production by cells such as dental pulp fibroblasts, which respond to invading microorganisms and produce chemokines. In dental pulp, the elevation of MCP-1 that is a chemo-attractant, gathers immune cells (to resist infection), which release more pro-inflammatory mediators and inflammatory cells within the tissues [29,30].

Recently, some lipoproteins from gram-positive bacteria were found to be triacylated, which are native ligands of TLR2 [31]. TLR2, along with other pattern recognition receptors (PRRs), are functionally the predominant receptors that stimulate production of the inflammatory mediators, IL-8, IL-6, MCP-1, and PGE2. Therefore, these receptors play important roles in the immune response of the dental pulp, and the progression of pulpitis [32]. Their expression in hDPCs alone appears to upregulate pro-inflammatory mediators. Likewise, our prior study reported that *S. gordonii* induces nitric oxide production through the TLR2 signaling pathway, and lipoprotein is responsible for the induction [7].

Now, this study has shown that the upregulated expression of inflammatory mediators in hDPCs that have been stimulated with *S. gordonii* lipoprotein can be attenuated by either CH or HBD3-C15. Such a capacity to reduce pulpal inflammation, especially in regenerative endodontic procedures on immature teeth may be particularly beneficial. The anti-inflammatory effects of CH are less understood than its known antimicrobial activity. CH inactivates the LPS of gram-negative bacteria, by hydrolyzing fatty acids in the lipid A moiety [9,33,34,35]. Additionally, it inactivates the LTA of gram-positive bacteria by deacylation, which inhibits their binding of TLR2 [10,11]. However in this study CH and HBD3-C15 suppressed lipoprotein-induced inflammation without blocking TLR2 signaling. It is speculated that the inflammation control of CH and HBD3-C15 as an endodontic medicament did not affect immune related signals in bacterial virulence factor-induced inflammation.

In this study, definite alleviation of the upregulated COX-2 as well as increased PGE2 secretion were observed in after CH- or HBD3-C15-treated hDPCs. Considering that PGE2 could enhance pain transmission in neuro-inflammation [36], the medication-dictated modulation of PGE2 release may provide a plausible background for why CH has been advocated for wide use in endodontics, and also adduce the possibility of HBD3-C15 as an alternative medicament. COX-2 is a membrane associated enzyme that produces PGE2 at sites of pulpal injury and inflammation, which leads to tissue swelling, redness, and pain [37]. As COX-2 participates in the regulation of prostanoid formation in the pathogenesis of pulpal inflammation, it was suggested that COX-2 may play a pivotal role in generating high levels of PGE2 locally resulting in pulpal tissue destruction, which formed the first investigation into using COX-2 inhibitors to control pulpal inflammation [38]. It is necessary to further investigate the effects of CH and HBD3-C15 on differential signal transduction pathways that mediate COX-2 stimulation and PGE2 production in hDPCs, instead of direct effect on TLR2 pathway. Another important clinical factor of the two chemicals would be a topical action in the control of inflammation, which could reduce the side effects of systemic anti-inflammatory drugs (i.e., NSAIDs). However, further studies are necessary to decipher the details of this mechanism.

The anti-inflammatory effects of HBD3-C15 peptide that were found in TLR-2 mediated inflammation in this study, were not in accordance with prior reports on the full length HBD3 protein pathways [16,39,40]. Just as other AMPs that permeabilize microbial membranes and neutralize or disaggregate LPS [16], full length HBD3 can bind directly to LPS and prevent the binding of LPS to host cell receptors [16,39,40] through TLR-4 mediated signaling pathways and the subsequent transcriptional inhibition of inflammatory genes [41]. It has also been suggested that full length HBD3 has anti-inflammatory properties that do not involve direct peptide binding to LPS, in macrophages isolated from human bone marrow [40]. However, this therapeutic application of full length HBD3 is limited by its molecular size, the complexity of disulfide pairing, and attenuated activity at elevated ionic strength. To overcome these limitations and identify the active peptide fragments within HBD3, the C-terminal HBD3 peptide was modified by substituting serine for cysteine residues, and shown to have retained its anti-microbial activity [42]. Recent reports showed that HBD3-C15 peptide could attenuate LPS-induced bone resorption, by disrupting podosome belt formation in osteoclasts and suppressing their differentiation [43]. In anti-inflammatory effect, HBD3-C15 did not affect upregulated TLR-2 level and its following signaling pathways in this study. This may have been due to the study design where CH paste and HBD3-C15 treatments were not applied simultaneously with *S. gordonii* lipoprotein. This was important to avoid the possible impairment or denaturation of lipoprotein and subsequent inflammatory mediators by CH or HBD3-C15, and to focus solely on the changes in lipoprotein-stimulated hDPCs.

Collectively, the results of this study support the use of CH and HBD3-C15 as intracanal medicaments, which may be particularly important for regenerative endodontic procedures in immature necrotic teeth. The synthetic HBD3-C15 peptide has multiple properties that are beneficial for its therapeutic application as an intracanal medicament in recurrent apical periodontitis and also in regenerative endodontic procedures for immature necrotic teeth.

## 4. Materials and Methods

### 4.1. Human Dental Pulp Cell Explant Cultures

Human dental pulp cell (hDPCs) explants were grown from the pulps of extracted teeth, which had been approved for collection by the Institutional Review Board of Seoul National University (S-D2014007, 27 March 2014). Crowns of freshly extracted teeth were split open and their pulps carefully harvested. The pulp tissues were diced into fine fragments in culture dishes (Nalge Nunc International, Rochester, NY, USA), and incubated in DMEM (Dulbecco’s modified Eagle’s medium) supplemented with 10% fetal bovine serum (FBS) and 1% penicillin-streptomycin, with fresh media replenished every 3 days. After 3 weeks of growth, the cells that had extended out from the tissue fragments were carefully harvested and sub-cultured. Following 4–6 passages, cells were utilized in experiments such that each independent experiment was performed with cells from the same passages.

**Figure 5:**
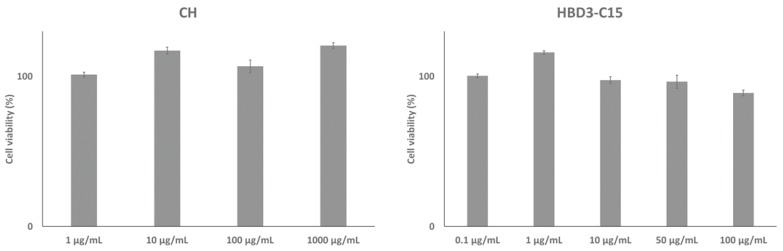
The survival rate of hDPCs was assessed in the presence of calcium hydroxide (CH) and HBD3-C15 using a cell counting kit (CellCountEZ^TM^ Cell Survival assay kit, Rockland Immunochemicals, Posttown, PA, USA). Briefly, the hDPCs were seeded in a 96-well plate at a density of 2 × 10^3^ cells/well and cultured with different concentrations of reagents for 24 h. The absorbance was measured with a microplate reader (Bio-Rad Laboratories, Hercules, CA, USA) at 490 nm.

The viability of hDPCs was quantitatively assessed in the presence of CH and HBD3-C15 using a cell counting kit (CellCountEZ^TM^ Cell Survival assay kit, Rockland Immunochemicals, Posttown, PA, USA). Briefly, the hDPCs were seeded in a 96-well plate at a density of 2 × 10^3^ cells/well and cultured with different concentrations of reagents for 24 h. The absorbance was measured with a microplate reader (Bio-Rad Laboratories, Hercules, CA, USA) at 490 nm (Figure 5).

### 4.2. S. gordonii Lipoprotein Extracts

Bacterial lipoproteins were isolated and purified from *S. gordonii*, as previously reported [44]. Briefly, bacterial pellets were collected and suspended in Tris-buffered saline (TBS) with protease inhibitors. They were sonicated, the lysates suspended in a final concentration of 2% Triton X-114 at 4 °C for 2.5 h, and then centrifuged to discard cell debris. The supernatant was incubated at 37 °C for 15 min and centrifuged again to separate the Triton X-114 phase from the aqueous phase which was discarded. An equal volume of TBS was added to the Triton X-114 phase, incubated at 37 °C for 15 min, and centrifuged again to discard the aqueous phase. Finally, the Triton X-114 phase was incubated overnight with methanol at −20 °C, and lipoprotein precipitates collected and dissolved in 10 mM octyl-beta-D-glucopyranoside in PBS.

### 4.3. Cell Transfection

HEK293-TLR2 cells were transfected as previously described [45]. Briefly, HEK293-TLR2 cells (2.5 × 10^5^ cells/mL, 5 mL, 60 mm dishes) were transfected with an NF-κB (nuclear factor-κB) reporter gene construct (pNF-κB-Luc, Clontech, Mountain View, CA, USA). Transfections were performed in Opti-MEM using Attractene transfection reagent for 16 h (Qiagen, Germantown, MD, USA). After collection, the cells (2.5 × 10^5^ cells/mL, 200 μL, 96-well plates) were plated in complete DMEM. The cells were then stimulated with *S. gordonii* lipoprotein and lysed with GloLysis Buffer (Promega, Madison, WI, USA). The cytoplasmic extracts were assayed with a luminometer (Molecular Devices, Sunnyvale, CA, USA) for luciferase activity.

### 4.4. Peptide Preparation

Peptides were synthesized at the central research institute of NIBEC using a peptide synthesizer (Prelude, Protein Technologies Inc., Tucson, AZ, USA) to produce the C-terminal amide form using standard 9-fluorenylmethoxycarbonyl (F-moc) chemistry and purified using preparative reverse-phase high-performance liquid chromatography (RP-HPLC; Waters, Milford, MA, USA) with a Vydac C18 column and a 50-min gradient from 90% to 10% water/acetonitrile containing 0.1% trifluoroacetic acid (TFA). The purity of the peptides was determined using HPLC (Shimadzu, Kyoto, Japan) and liquid chromatography-mass spectrometry (LC-MS, Shimadzu, Kyoto, Japan). The purity of the peptide was measured as 99.3% and the molecular weight of the peptide was identified as 1766.17 (Da) using LC-MS.

### 4.5. Inflammatory Chemokine Analysis

The hDPCs (2.5 × 10^5^ cells/mL) were plated in six well plates and stimulated with 10 μg/mL of lipoprotein extracts for 24 hrs. The stimulated cells were then treated with either CH (DC Chemical Co Ltd., Seoul, Korea) mixed with distilled water (100 µg/mL) as the control, or synthetic HBD3-C15 peptide gel (50 µg/mL). The synthetic HBD3-C15 peptide (NIBEC, Seoul, Korea) was prepared by F-moc-based chemical solid phase synthesis from 15 amino acids (GKCSTRGRKCCRRKK). It was used at a concentration (50 µg/mL) that was found to be effective in a previous study [14].

Time points were chosen that included before and after 24 h of lipoprotein stimulation, as well as after 1, 3, and 7 days of the treatments with either CH or HBD3-C15. At these predetermined times, replicate (N = 3) hDPC cultures were harvested and their total cell RNA extracted with RNAiso Plus reagents (Takara, Otsu, Japan). The cDNA was synthesized with gene-specific reverse primers for humans (Table 1) by using the PrimeScript RT reagent kit (Takara). Reverse transcription-quantitative polymerase chain reaction (RT-qPCR) was done with a C1000 Real-time PCR system thermal cycler (Bio-Rad, Hercules, CA, USA). The level of the target gene transcripts (*Δ*CT) was normalized to that of glyceraldehyde-3-phosphate dehydrogenase (Gapdh). The relative levels of expression in the experimental group were calculated by *Δ*(*Δ*CT) (*Δ*CT_Control_ − *Δ*CT_Experiment_). The findings were verified by multiplex analysis according to the manufacturers’ instructions. Data was collected using the Luminex-100 system (ver. 1.7, Luminex, Austin, TX, USA) and analyzed by using the Milliplex Analyst (Viagene Tech, Carlisle, MA, USA). A five-parameter regression formula was used to calculate the concentrations from the standard curves. Reported data as normalized pg/mL was assessed. All reactions were run in triplicate.

### 4.6. Statistical Analysis

Replicate cultures were compared both before and after lipoprotein-stimulation, and then after treatment with either calcium hydroxide or HBD3-C15 for 1, 3, and 7 days. Data were analyzed statistically by a one-way analysis of variance (ANOVA) and Tukey’s post-hoc test to a significance of *P* < 0.05.

## Figures and Tables

**Table 1:** Sequences of primers used in RT-qPCR.

Gene (Human)	Primer (5′→3′)	
*IL-8*	Forward	AGGGTTGCCAGATGCAATAC
	Reverse	CCTTGGCCTCAATTTTGCTA
*MCP-1*	Forward	GCAGCAAGTGTCCCAAAGA
	Reverse	ACAGGGTGTCTGGGGAAAG
*COX2*	Forward	TTCAAATGAGATTGTGGGAAAATTGCT
	Reverse	AGATCATCTCTGCCTGAGTATCTT
*TLR2*	Forward	CCCATTGCTCTTTCACTGCT
	Reverse	CTTCCTTGGAGAGGCTGATG
*GAPDH*	Forward	GGCTGAGAACGGGAAGCTT
	Reverse	TCCATGGTGGTGAAGACGC
